# The Potential of Wearable Sensors for Detecting Cognitive Rumination: A Scoping Review

**DOI:** 10.3390/s25030654

**Published:** 2025-01-23

**Authors:** Vitica X. Arnold, Sean D. Young

**Affiliations:** 1 Department of Informatics, University of California, Irvine, CA 92697, USA; vxarnold@uci.edu; 2 Department of Emergency Medicine, University of California, Irvine, CA 92697, USA

**Keywords:** cognitive rumination, perseverative cognition, wearable devices, wearable technology, mental health monitoring, biosensors, psychophysiology, mental health technology, stress detection, anxiety

## Abstract

Cognitive rumination, a transdiagnostic symptom across mental health disorders, has traditionally been assessed through self-report measures. However, these measures are limited by their temporal nature and subjective bias. The rise in wearable technologies offers the potential for continuous, real-time monitoring of physiological indicators associated with rumination. This scoping review investigates the current state of research on using wearable technology to detect cognitive rumination. Specifically, we examine the sensors and wearable devices used, physiological biomarkers measured, standard measures of rumination used, and the comparative validity of specific biomarkers in identifying cognitive rumination. The review was performed according to the Preferred Reporting Items for Systematic reviews and Meta-Analyses (PRISMA) guidelines on IEEE, Scopus, PubMed, and PsycInfo databases. Studies that used wearable devices to measure rumination-related physiological responses and biomarkers were included (*n* = 9); seven studies assessed one biomarker, and two studies assessed two biomarkers. Electrodermal Activity (EDA) sensors capturing skin conductance activity emerged as both the most prevalent sensor (*n* = 5) and the most comparatively valid biomarker for detecting cognitive rumination via wearable devices. Other commonly investigated biomarkers included electrical brain activity measured through Electroencephalogram (EEG) sensors (*n* = 2), Heart Rate Variability (HRV) measured using Electrocardiogram (ECG) sensors and heart rate fitness monitors (*n* = 2), muscle response measured through Electromyography (EMG) sensors (*n* = 1) and movement measured through an accelerometer (*n* = 1). The Empatica E4 and Empatica Embrace 2 wrist-worn devices were the most frequently used wearable (*n* = 3). The Rumination Response Scale (RRS), was the most widely used standard scale for assessing rumination. Experimental induction protocols, often adapted from Nolen-Hoeksema and Morrow’s 1993 rumination induction paradigm, were also widely used. In conclusion, the findings suggest that wearable technology offers promise in capturing real-time physiological responses associated with rumination. However, the field is still developing, and further research is needed to validate these findings and explore the impact of individual traits and contextual factors on the accuracy of rumination detection.

## 1. Introduction

Rumination, a complex concept in psychological literature, is broadly understood as a cognitive process of repetitive, often negative thinking that can be distressing and intrusive [1,2]. Rumination can be measured in two forms: state rumination and trait rumination. Trait rumination refers to an individual’s predisposition or tendency to engage in ruminative thinking as a stable personality trait. Individuals with high levels of trait rumination are more likely to ruminate frequently in their daily lives, regardless of what events transpire [3,4,5]. In contrast, state rumination refers to a temporary ruminative episode responding to a stressful or negative event [3,6]. Rumination is considered a transdiagnostic symptom, meaning it is a symptom observed across multiple mental health conditions [7]. Distinct from mental health disorders themselves, rumination functions as a symptom within a broader clinical context rather than as an independent diagnosis [1,2,7]. As a form of perseverative cognition [8], rumination shares characteristics with other repetitive thought patterns like worry [1]. Although both rumination and worry involve repetitive thinking, they are distinct. Rumination typically centers on dwelling on past negative thoughts or experiences, whereas worry tends to focus on concerns about future uncertainties [1]. These distinctions highlight rumination’s role in understanding cognitive responses related to stress and past negative experiences [9,10,11].

While no technology or standardized clinical protocols are specifically designed for using biomarkers to detect cognitive rumination, emerging research has identified physiological and neurological biomarkers related to rumination that could guide the development of such technology in the future [12,13,14]. Among the most significant associations are EDA and cardiovascular biomarkers, both of which are influenced by the activation of the sympathetic nervous system. State dysphoric rumination has been shown to activate the sympathetic nervous system, which regulates processes such as heart rate, blood pressure, and sweat production (i.e., skin conductance) [15]. These physiological changes are reflected in measures—HRV, skin conductance, muscle responses, and movement—that can be captured by sensors. Numerous studies have found that rumination induction leads to increased skin conductance and heart rate and reduces HRV [16,17,18], which is linked to various adverse health outcomes [19,20,21]. Neurological biomarkers of rumination have been associated with the brain’s default mode network (DMN) and prefrontal regions. Structural and functional abnormalities have been identified in the precuneus, a central DMN node, which is linked to reduced brain coherence and repetitive, maladaptive thought patterns [22,23]. Moreover, increased activity in the dorsolateral prefrontal cortex has been associated with state rumination, especially in individuals with depression [6]. While specific brain waves uniquely tied to rumination are not yet fully established, early evidence links rumination to distinct patterns of electrical activity across brain regions. For instance, enhanced slow-frequency power in the precuneus and reduced high-gamma power in the hippocampus may reflect neural correlates associated with rumination [24]. It is important to note that many of these biomarkers are not exclusive to rumination and may overlap with other mental health disorders. Eventually, the combination of sensor data collected from biomarkers, such as increased skin conductance and altered DMN activity, may enable more accurate detection of rumination.

Rumination, a perseverative thinking process focused on negative content, significantly impacts mental and physical health across diverse demographic and clinical contexts. For this scoping review, we adopt Sansone and Sansone’s 2012 definition of rumination as “a detrimental psychological process characterized by perseverative thinking around negative content that generates emotional discomfort” [25]. Ruminative episodes vary across gender and age, with women generally engaging in more ruminative thinking than men [26,27] and ruminative thinking peaking in young adulthood before declining over the lifespan [28]. These demographic patterns in rumination are particularly significant given that rumination manifests as a transdiagnostic symptom across mental health conditions, including depression, generalized anxiety, post-traumatic stress disorder, social anxiety disorder, obsessive–compulsive disorder, bipolar disorder, and psychosis spectrum disorder [7,29,30,31,32,33]. Its effects on both mental and physical health are profound, exacerbating existing psychological disorders through various mechanisms [34] while contributing to physiological dysregulation and the development of chronic health conditions, particularly chronic pain [4,35,36]. Research indicates that rumination is most frequently triggered by interpersonal interactions and negative experiences, with many individuals reporting difficulty inhibiting these perseverative thought patterns once initiated [37].

The current clinical method for assessing rumination is through self-report questionnaires. The Response Styles Questionnaire—Rumination Response Scale (RRS), introduced in 1991 [38], serves as the foundational measure in this field. While subsequent measures like the Rumination Questionnaire [39], the Event Related Rumination Inventory [40], and the Pain Catastrophizing Scale [41] have expanded our ability to capture different aspects of rumination, from general cognitive processes to pain-specific rumination, these traditional assessment methods share fundamental constraints. While individuals might ruminate on negative content ranging from interpersonal conflicts and perceived personal shortcomings to deeper existential uncertainties, commonly used rumination questionnaires do not differentiate between these distinct categories. Instead, they primarily focus on broader aspects of rumination through two main subscales: brooding and reflection [42]. Beyond this limitation in categorization, the reliance on retrospective self-reporting makes traditional rumination assessments susceptible to recall and social desirability bias, as participants must accurately remember and report their ruminative episodes [43]. Furthermore, these instruments only capture data at specific points in time, potentially missing the dynamic nature of rumination as it fluctuates throughout daily life. This fixed-point data collection cannot capture the moment-to-moment variations in ruminative thinking that may be crucial for understanding its true impact on mental health.

Wearable technology includes a wide array of small, portable electronic devices that are worn on the body or implanted and are designed to collect and transmit data. These devices are used across multiple sectors, including consumer electronics, healthcare, and research. They typically connect to smartphones, which handle data processing and enable communication [44]. For this review, we follow [44]’s definition, which describes wearable devices as “small electronic and mobile devices, or computers with wireless communications capability, embedded in gadgets, accessories, or clothing, that can be worn on the body”. In mental health research, these technologies are categorized into commercial, research, and medical-grade devices, each offering distinct levels of accessibility, regulatory standards, and data precision [45,46,47,48]. However, there is a notable trend of repurposing consumer devices for mental health research despite their original fitness-tracking intent [45]. Furthermore, wearable technologies in health monitoring can be broadly categorized into invasive and non-invasive devices, with invasive methods involving bodily insertion and presenting inherent risks and comfort challenges in exchange for their data precision [49]. In contrast, non-invasive devices, which collect data from the body’s surface without penetration, offer essential health insights while prioritizing user comfort, accessibility, and reduced medical complications [50,51]. Given the advantages of non-invasive wearables and the growing availability of wearable technology for health research, this scoping review focuses exclusively on non-invasive devices. In mental health research, wrist-worn devices equipped with sensors to measure physiological biomarkers are the most common wearable device employed [52], with HRV emerging as a key metric for stress and anxiety detection [45]. The field continues to explore the potential of less common wearable forms and sensor types (i.e., smart glasses, belts, necklaces, and clips) for comprehensive mental health monitoring [52].

Wearable technologies might improve measuring cognitive rumination by continuously capturing physiological indicators in real-world settings. Capturing data between self-report time periods enhances ecological validity and research scalability. Specifically, these devices monitor physiological indicators associated with stress and negative affect, such as HRV, EDA, and skin temperature, which research has shown to correlate with ruminative thought patterns [17,45,50]. The advantages of wearable technology for rumination research extend beyond physiological monitoring, including non-invasive data collection, cost-effectiveness, and enhanced accessibility, potentially transforming our understanding of and intervention in ruminative thoughts in naturalistic settings [48,53,54,55,56]. However, significant challenges persist, including data quality, reliability concerns, battery life limitations, digital equity issues, and ethical considerations surrounding the collection and storage of sensitive personal health data [44,57,58,59].

A scoping review assessing the use of wearable technology to detect cognitive rumination is needed in the current landscape of mental health research and technology. While rumination is a prevalent and impactful transdiagnostic symptom affecting a significant portion of the population, traditional assessment methods remain limited by their reliance on self-reporting and their fixed point-in-time data collection. The rapid advancement of wearable technology has the potential for continuous, ecologically valid monitoring of physiological indicators associated with rumination. However, to the best of our knowledge, very few literature reviews, if any, have been conducted on the intersection of wearable technology and cognitive rumination detection. To this end, we examine the existing research studies using wearable technology to detect cognitive rumination, sensors and wearable devices used, physiological biomarkers measured, standard measures of rumination used, and the comparative validity of specific biomarkers in identifying cognitive rumination, which are reported in this review.

## 2. Materials and Methods

The method that we employed for the research design was that of a scoping review, following both Arksey and O’Malley’s framework and the PRISMA guidelines [60,61,62]. This process consists of 5 main steps: identifying the research questions, identifying relevant studies, identifying inclusion criteria, charting the data, and lastly, summarizing and reporting the results [61].

### 2.1. Identifying the Research Questions

This paper presents a scoping review of current research studies related to cognitive rumination. The review aims to answer the following research questions:

- RQ1: What existing research studies have used wearable technology to measure or detect cognitive rumination?
- RQ 2: What sensors and wearable devices are used to collect physiological markers of rumination, and which specific physiological markers are measured using these devices?
- RQ3: What standard measures of rumination are being used in rumination studies involving wearable devices?
- RQ4: What is the comparative validity of physiological biomarkers in identifying rumination?

### 2.2. Identify Relevant Studies

To ensure a comprehensive and focused literature search, we employed a multi-step process. Initially, we conducted a preliminary manual search to identify eligible studies and determine relevant databases and query terms. This preliminary process revealed an unexpected challenge: the term “rumination” introduced noise to the corpus due to its association with cattle, sheep, and other grazing animals. In the context of livestock, rumination describes a regurgitative digestive process common among these animals [63]. To address this issue, we refined our search strategy by adding the Boolean string “AND NOT (“livestock” OR “cattle” OR “sheep” OR “cow” OR “dairy”)” to exclude sources related to grazing animals.

The main literature search was then conducted across four databases: IEEE, Scopus, PubMed, and PsycInfo. To further enhance the comprehensiveness of our search, a manual citation search was conducted using reference lists of relevant studies to identify other potentially eligible studies. The detailed search strategies used to find relevant studies for this scoping review are described in Table 1.

### 2.3. Selection of Eligible Studies

The researchers followed the steps outlined in the PRISMA guidelines to select eligible studies [61,62]. First, the titles and abstracts were screened and filtered. Then, we conducted a full read of the remaining corpus. The following inclusion and exclusion criteria were used to determine relevant studies:

The inclusion criteria were as follows:

- Published in English;
- Peer-reviewed publication;
- Full text was available;
- Studies that used non-invasive wearable devices (the term non-invasive refers to devices that collect data without penetrating the body or causing physical discomfort to the subject);
- Studies that use one or more wearable sensors or devices to measure physiological indicators related to rumination.

The exclusion criteria were as follows:

- Studies published in other languages;
- Patents, books, editorial papers, dissertations, news articles, letters, notes, surveys, and erratum.

### 2.4. Data Charting

Guided by the research questions, the following details were extracted from the selected studies:

- The aims of each study;
- The year each study was published;
- Number of participants for each study;
- The name and brand of each wearable device used to collect physiological data;
- The types of wearable devices used to collect physiological data related to rumination;
- The sensor type and their physiological biomarkers used to measure rumination;
- Any additional assessments of rumination that were used in combination with physiological measurements;
- The usability and feasibility of each device, including any factors that hindered or facilitated their use, if any;
- Notes of data quality of each device, if any.

### 2.5. Collating, Summarizing, and Reporting the Results

Results collected from the selected studies were then presented in a narrative format. To answer the research questions, the results are organized into six sections: data charting, an overview of selected studies, physiological biomarkers, and their sensors, types of wearable devices, study summaries, rumination measures, and comparative biomarker validity for rumination detection.

The rest of the review is structured as follows: Section 3 presents the results, starting with the PRISMA figure to identify the literature corpus, followed by a breakdown of selected articles by year of publication. Section 3.1 provides a summary of the key characteristics of the selected studies. Section 3.2 examines physiological biomarkers, the associated sensors, and their definitions. Section 3.3 discusses device types, models, and the corresponding studies. Section 3.4 summarizes the studies and categorizes them by context. Section 3.5 reviews the measures of rumination and the induction protocols used. Section 3.6 assesses the comparative validity of biomarkers for detecting rumination. Section 4 presents the discussion, starting with an in-depth analysis of the studies, including their relationships, similarities, and differences. Section 4.1 explores the opportunities and challenges in capturing real-time physiological responses to rumination, while Section 4.2 situates wearable research within the broader field of rumination studies and highlights future research directions.

## 3. Results

**Figure 1:**
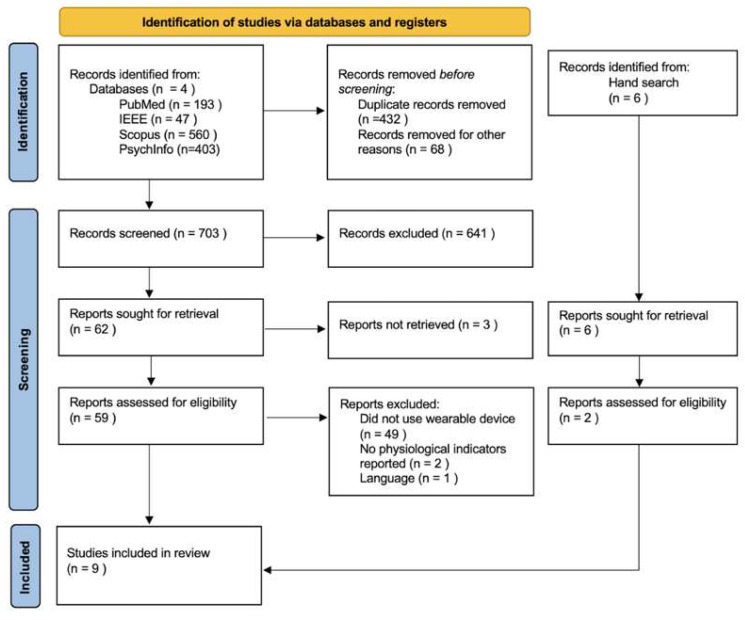
Presents the article selection process flowchart modified from the suggested template in PRISMA.

A total of 1203 articles were identified, out of which 68 were filtered out by the database platform for being patents, books, editorial papers, dissertations, news articles, letters, notes, surveys, or erratum. Of the remaining articles, 432 duplicates were removed. After title and abstract screening, 641 articles were excluded because they did not meet the predefined eligibility criteria, and three articles were unable to be retrieved. As a result, 59 articles were read in full, and 52 of these were excluded. The remaining seven studies were included with two additional manual searches, resulting in the nine studies shown in Figure 1.

**Figure 2:**
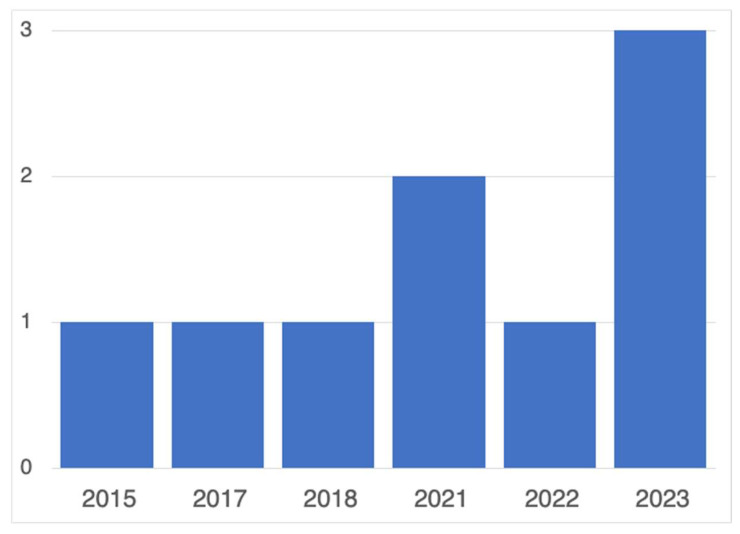
Presents the categorization of the selected articles by year published.

Given the limited corpus observed in other reviews related to wearables and anxiety [52], we anticipated a small final corpus for this review. As a result, we did not impose any time constraints during the review process. The earliest study in our corpus using wearable technology dates to 2015, which aligns with the broader adoption of wearable technology in healthcare around 2014 [64]. The studies were then categorized by year of publication, as shown in Figure 2.

### 3.1. Overview of Selected Studies on Rumination Detection

The following section as described in Table 2 provides an at-a-glance summary of the selected studies in our final literature corpus to give a comprehensive overview of each study’s scope and devices used. Specifically, the table lists the article titles, the brand names and models of each wearable device used, the types of sensors used in these devices, and the biomarkers measured. Additionally, it includes details about supplementary assessments used for evaluating rumination, the specific aims of each study, the sample sizes of each study, and their corresponding references. 

### 3.2. Physiological Biomarkers and Their Associated Sensors

For clarity in navigating this review, it is important to note that researchers use several interchangeable terms when discussing skin conductance measurements, including EDA, skin conductance level (SCL), skin conductance response (SCR), and galvanic skin response (GSR). EDA has accumulated numerous names throughout its research history, reflecting the diverse scientific disciplines that have studied this physiological phenomenon. Researchers have used various terms such as GSR, psychogalvanic reflex (PGR), and electrodermal response (EDR) to describe these electrical properties of the skin. According to [74], the scientific community has now standardized the terminology to EDA, which serves as an umbrella term for measuring both tonic and phasic changes in skin conductance. When measuring tonic activity, EDA records the SCL, which represents slower-changing baseline conductance levels [75,76]. For phasic activity, EDA measures SCR, which captures rapid conductance changes in response to specific stimuli [75,76]. This standardization helps address the fragmentation in terminology, though some older terms like GSR remain in use, particularly in legacy literature. In this review, we use the original terms chosen by each study’s authors to accurately reflect their work while recognizing that these different terms describe the same physiological measure.

**Figure 3:**
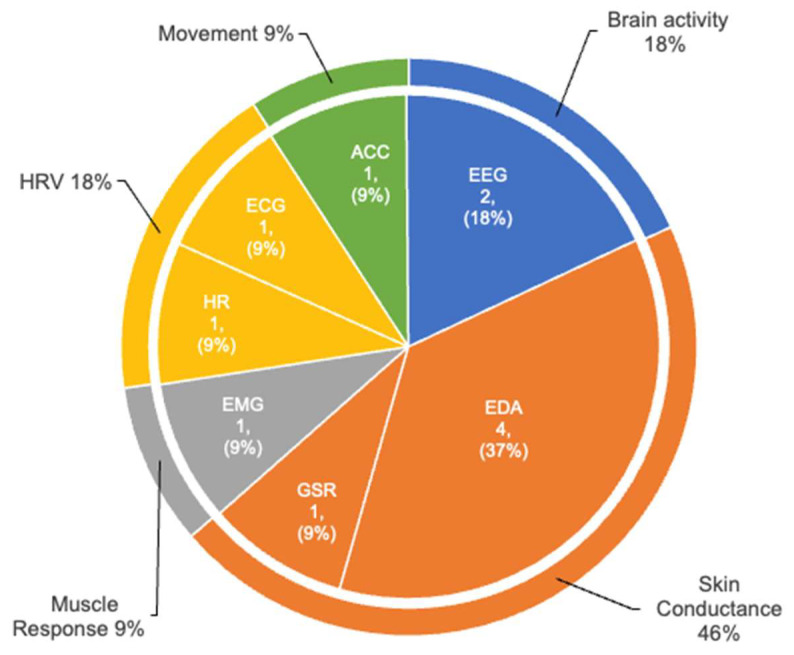
Presents the distribution of physiological biomarkers (outer donut chart) and their corresponding sensor types (inner pie chart) across our corpus (*n* = 9). While most studies measured a single biomarker (*n* = 7), two studies measured multiple biomarkers (*n* = 2). The biomarkers represented in our final corpus include skin conductance (*n* = 5, 46%), brain activity (*n* = 2, 18%), movement (*n* = 1, 9%), HRV (*n* = 2, 18%), and muscle response (*n* = 1, 9%). Sensors used were EDA (*n* = 4, 37%) and GSR (*n* = 1, 9%) for skin conductance, EEG (*n* = 2, 18%) for brain activity, accelerometers (*n* = 1, 9%) for movement, ECG (*n* = 1, 9%) and HR monitors (*n* = 1, 9%) for HRV, and EMG (*n* = 1, 9%) for muscle response.

Figure 3 depicts the prevalence of various physiological biomarkers and their corresponding sensor types used to measure or detect cognitive rumination through wearable devices in our corpus. The pie chart reveals several notable trends: (1) Skin conductance, measured using EDA or GSR sensors, emerges as the most prevalent physiological biomarker. These EDA measurements include both SCR and SCL. (2) Electrical brain activity, measured through Electroencephalogram (EEG) sensors, ranks as the second most prevalent physiological biomarker. (3) HRV, measured using Electrocardiogram (ECG) sensors and HR fitness monitors, represents the third most prevalent physiological biomarker. (4) Movement and muscle response, measured using accelerometer (ACC) sensors and Electromyography (EMG) sensors, respectively, are the least prevalent physiological biomarkers in the corpus.

### 3.3. Wearable Devices for Rumination Detection

All the studies in this review used a form of wearable device. Table 3 summarizes the different types of non-invasive wearable devices and shows how frequently each type was used across the studies.

The RS-800CX Polar Electro and Bodyguard 2 Firstbeat was used in [65]. Trigno Mini sensors and Trigno wireless EMG system were used in [66]. Neulog GSR logger sensor NUL-217 was used in [67]. Empatica E4 was used in [68,71], and Embrace 2 was used in [69]. Emotiv Insight portable headset was used in [70]. Wireless EEG ZhenTec NT1 was used in [72]. MindWare Mobile Acquisition Unit was used in [73].

### 3.4. Existing Studies on Measuring Rumination Using Non-Invasive Wearables

These nine studies examine rumination’s physiological correlates, its effects on mood and autonomic function, and its interactions with factors such as stress, suicidal thoughts, and mindfulness across diverse populations and contexts. They investigate topics including rumination’s link to depression and suicidal ideation in clinical populations, the impact of race-related stress on minoritized students, and its role in stress recovery and mindfulness in daily life. The studies employ wearable sensors alongside methods such as ecological momentary assessments (e.g., electronic diaries), traditional self-report tools, and natural language processing. Overall, we identified two clear consistencies across the studies. First, there was strong methodological consistency, with most studies relying on standardized rumination scales and induction protocols derived from Nolen-Hoeksema’s foundational work. Second, a multimodal approach was commonly used, as many studies combined wearable sensor data with self-reported measures to enhance validity and capture aspects of rumination a single method might miss. While there was heterogeneity in the types of biomarkers assessed, the consistent findings of increased EDA, decreased HRV, and changes in EEG activity provide a starting point for further research into the potential of wearable sensors for detecting cognitive rumination. This section is organized by context, beginning with studies on rumination, then its role in depression and suicidal ideation, and concluding with its relevance to Ecological-Based Mindfulness.

#### 3.4.1. Rumination Among the General Population

The authors of [66] investigated the use of wearable EMG devices to measure verbal rumination, a form of inner speech or internal dialogue where someone talks to themselves mentally rather than out loud. The study involved 72 female undergraduate students who were first assessed for their tendency to ruminate in daily life using the French version of the Mini Cambridge-Exeter Repetitive Thought Scale (Mini-CERTS) [77]. Thereafter, participants underwent a negative mood induction using a validated forced-failure task that involved a nearly impossible-to-solve timed IQ test. Following the negative mood induction, participants spent 5 min engaging in rumination using prompts adapted from Nolen-Hoeksema’s [78] rumination induction paradigm. They were asked to silently reflect on the causes and consequences of their feelings. During the final minute of the rumination period, researchers measured muscle activation using EMG Trigno Mini Sensors across three distinct regions: the speech-related labial muscles comprising the Orbicularis Oris Superior (OOS) and Orbicularis Oris Inferior (OOI), the negative-affect-related Facial Muscle Frontalis (FRO), and the flexor carpi radialis (FCR) in the forearm, which served as a non-facial control muscle. After this, participants were instructed to self-report momentary rumination using four visual analog scales (rated 0–100). These scales assessed different aspects of rumination: thoughts about feelings (VAS “Feelings: At this moment, I am thinking about my feelings”), focus on problems (VAS “Problems: At this moment, I am thinking about my problems”), brooding about negative things (VAS “Brooding: At this moment, I am brooding about negative things”), and self-focus (VAS “Focused: “At this moment, I am focused on myself”). Results revealed that even when completely silent, ruminative thoughts lead to increased activity in lip and forehead muscles. This corresponded with increased self-report rumination levels and was evident when participants prompted to ruminate showed increased muscle activity in speech-related muscles, including the forehead (FRO: α = 197.55, d = 0.74), lips (OOS: α = 138.57, d = 0.66), and jaw (OOI: α = 163.89, d = 0.77). This finding aligns with the Motor Simulation theory, which proposes that our internal dialogue engages our physical mechanisms of speech. Researchers further validated this connection by implementing relaxation exercises focused on the facial and mouth muscles. These exercises not only reduced unconscious muscle activity but also decreased participants’ self-reported rumination, highlighting a bidirectional relationship between physical muscle tension and mental thought patterns. The results suggest that labial EMG could be a valuable objective tool for assessing ruminative inner speech.

The authors of [73] compared the effects of imagery and verbal-based rumination and distraction on adolescents’ psychophysiological responses. The study involved 145 adolescent participants who first underwent negative mood induction using Cyberball, a validated psychological task designed to induce negative affect through perceived peer rejection in a virtual ball-tossing game [79]. Following negative mood induction, participants were assigned to either rumination or distraction conditions, implemented through either mental imagery or verbalization. The rumination induction involved 24 prompts adapted from Nolen-Hoeksema’s [78] rumination induction paradigm (e.g., “Think about/imagine the kind of person you think you should be”), with each prompt displayed for 15 s, followed by one-minute rest periods. The distraction condition used similar timing but with neutral prompts (e.g., “Think about or imagine the items on your grocery list”) [73]. Researchers collected multiple types of data: affective ratings using a visual analog scale (SAD 1 to HAPPY 100) at four time points, high-frequency heart rate variability (HF-HRV), and SCR via non-invasive ECG and EDA sensors on the MindWare Mobile Acquisition Unit. Results revealed that rumination’s negative effects on affect and stress levels were similar regardless of whether participants engaged in imagery-based or verbal-based rumination, suggesting that the process of ruminating on negative thoughts is more critical than the form it takes. Physiological data from wearable sensors objectively supported this finding, showing overall negative physiological effects in both rumination conditions. Specifically, HF-HRV waveforms revealed lower HF-HRV levels in the rumination condition compared to the distraction condition. Additionally, SCR waveforms indicated higher arousal levels in the rumination condition compared to the distraction condition. These physiological patterns of reduced HRV and elevated SCR have been found to be associated with heightened stress and anxiety, which research has linked to various adverse health outcomes [19,20,21]. While waveform graphs visually demonstrated these relationships, it should be noted that formal statistical analyses were not conducted to examine the associations between SCR, HRV, and rumination levels. Nonetheless, both HF-HRV and SCR emerged as reliable, objective measures of rumination when captured through wearable sensors, offering potential biomarkers for ruminative thinking.

The authors of [67] examined the effects of worry, rumination, and distraction on recovery from stress, testing predictions from the metacognitive model—a framework that explains how people think about their own thinking processes. The study involved 54 undergraduate participants (38 women, 16 men, mean age 20) who underwent a baseline measurement of positive and negative affect schedule (PANAS) and of SCL followed by a Trier Social Stress Test (TSST) to induce stress. The TSST instructed participants to complete two 5 min tasks in front of a trained experimenter. First, they delivered a speech explaining why they were the ideal candidate for their dream job, believing it was being recorded for peer review. Then, they performed a mental arithmetic task, counting backward from 1022 by 13 s, while receiving intentionally negative feedback from the experimenter, such as impatient finger tapping. Both components were designed to create a stressful environment used to induce negative affect. Following the stress induction, participants were randomly assigned to one of three conditions: worry, rumination, or distraction. The rumination induction involved 10 prompts beginning with “why?” to focus participants’ attention on emotions and symptoms of oneself (e.g., “Why am I feeling the level of motivation I feel right now?”). The worry condition used 10 prompts focused on future dangers and threats (e.g., “What if you were unable to maintain your current lifestyle?”). The distraction condition involved word search puzzles as an active control task. Each condition lasted 8 min. A set of 10 prompts was presented via cue cards and required written responses from participants in the worry and rumination conditions. Researchers collected SCL using the Neulog GSR logger sensor NUL-217 and negative affect scores using the PANAS scale. These measurements were taken at six time points: baseline, during stress, post-emotion regulation, and at three 10 min intervals during the 30 min recovery period. To ensure task engagement, participants rated their ability to stay on task using a visual analog scale (VAS) from 0 to 100. Results revealed that participants in the rumination condition experienced delayed physiological recovery compared to those in the worry and distraction groups. Specifically, SCL in the rumination group did not decrease as observed in the worry and distraction groups. Instead, SCL in the rumination group gradually increased, with the most significant difference from the distraction group occurring at the final measurement, 30 min into the recovery period (SCL mean difference = 1.54 microsiemens, *p* = 0.01). This finding was supported by a one-way ANOVA showing a large effect size (F(1,34) = 4.66, *p* = 0.04, η^2^ = 0.12). Notably, these physiological differences were captured exclusively through SCL measures, with no corresponding changes in self-reported indicators. The observed discrepancy between physiological and self-reported measurements highlights both the complexity of measuring ruminative experiences and the limitations of single-method assessments. This inconsistency between self-report and physiological measures may reflect either fundamental measurement challenges or indicate that these different approaches capture distinct aspects of rumination. Given these considerations, a multi-method assessment approach becomes crucial for ensuring comprehensive data collection and robust research findings.

Ref. [68] investigated how psychological health traits moderate the relationship between race-related stress and sympathetic nervous system arousal in racial and ethnic minority college students. The study involved 100 participants (74 African Americans, 24 Latinx, and 1 Mixed race) in a novel protocol that allowed researchers to jointly assess exposure to race-related stress, interpersonal discrimination experiences, discrimination rumination, and vicarious discrimination. Upon enrollment, participants completed an intake survey covering sociodemographics, psychological characteristics, and prior discrimination experiences. Participants were then trained to use the Empatica E4 wrist wearable device worn on their non-dominant wrist to collect EDA data, along with smartphone-delivered daily diaries. The diary protocol involved morning and evening surveys sent via SMS at 8 am and 8 pm, respectively. The evening diary used a day reconstruction method [80], where participants were instructed to recall events and feelings throughout the day in 15 min intervals called “moments”. For each “moment”, participants answered specific questions, including whether they had engaged in racism-related rumination (e.g., “Over the course of the day, did you think about racial injustices and the mistreatment of people of color in the US?”). Researchers collected continuous EDA measurements, a physiological indicator of arousal, using the Empatica E4 wrist-worn wearable device, and linked these data to diary entries. Results revealed distinct physiological stress responses during periods of racism-related rumination, with individual differences in emotional traits playing a key role. Participants with high anger (*p* = 0.012) and anxiety traits (*p* = 0.006) showed significantly elevated arousal levels during these periods, while those with low anger and anxiety traits exhibited suppressed physiological responses (*p* > 0.05). These contrasting patterns suggest that personality traits moderate how the body responds to race-related rumination. This physiological signature of rumination was validated through both subjective and objective measures, with participants’ self-reported diary entries aligning with their concurrent EDA measurements. The combination of diary reports and physiological data demonstrated that rumination triggers both psychological distress and measurable physiological responses. These findings highlight the potential of wearable EDA sensors as an objective tool for detecting rumination’s impact in naturalistic settings, offering a valuable complement to traditional self-report methods.

The authors of [72] conducted a randomized controlled trial investigating how a novel NLP-based questionnaire could induce ruminative thinking in rumination-prone individuals. After validating the questionnaire’s effectiveness, they used a wireless ZhenTec NT1 device to measure participants’ EEG responses while they engaged with the rumination-inducing questions. The study began with 4591 participants from Shaanxi Police College in China (4526 men, 5 women). Researchers first administered the Chinese version of the RRS to all participants to identify individuals with high rumination tendencies. From these results, 607 participants with high rumination scores were selected for individual semi-structured interviews focused on their experiences with rumination. These interviews were recorded, transcribed, and analyzed using NLP techniques to develop 37 rumination-inducing questionnaire items. The questionnaire underwent expert review by three linguistics professors and was pilot-tested on a randomly selected group of 78 participants (40 high ruminators, 38 low ruminators). Following validation, researchers conducted EEG measurements on a separate group of 85 selected participants (56 high ruminators as the experimental group, 29 low ruminators as the control group) while they responded to both ruminative and neutral items. EEG coherence measurements across multiple frequency bands (theta, alpha, beta, and gamma) were taken both during resting state and while participants engaged with the questionnaire items. Results revealed three key patterns that demonstrate both the effectiveness of the NLP-based questionnaire and the value of EEG measurements captured through wearables in studying rumination. First, arousal rates calculated through the proportion of “yes” responses to “Does the above description induce you to have repeated/continuous recall?” for rumination items showed that high ruminators experienced significantly higher arousal rates when responding to the questionnaire’s ruminative items compared to the control group (*p* < 0.001). Second, EEG within-group analysis revealed that the coherence of the whole brain decreased in both ruminators and controls while engaging with rumination items compared to neutral items, with the decrease in coherence significantly greater in ruminators [F(1,90) = 9.445, *p* = 0.003]. Both findings validated the effectiveness of the NLP-derived questionnaire in successfully triggering ruminative thinking patterns. Third, despite the general reduction in overall brain coherence, between-group EEG analysis revealed that high ruminators exhibited increased coherence in the beta (t = 2.301, *p* = 0.017) and gamma (t = 2.411, *p* = 0.018) frequency bands during rumination-inducing questions. Previous research suggests these patterns may indicate diminished control over unwanted thoughts, potentially stemming from an excessive self-focused depressive state [81] and the recollection of negative memories that the rumination items elicited [82,83]. Fourth, Pearson correlation analysis revealed a significant relationship between arousal rates and EEG coherence, specifically in the gamma2 frequency band (r = 0.231, *p* = 0.046). This finding strengthens the connection between gamma2 activity and emotional responses to rumination, suggesting that increased coherence in the gamma2 band may serve as a neural indicator of the negative emotional states typically associated with ruminative thinking. These findings demonstrate that while EEG cannot directly measure thought content, coherence analysis of EEG data, particularly in the gamma2 frequency band, can effectively capture distinct neural signatures of rumination, suggesting its potential as a reliable biomarker for identifying and monitoring ruminative states.

#### 3.4.2. Rumination in the Context of Depression and Suicidal Ideation

The authors of [65] conducted a quasi-experimental study examining the autonomic concomitants of intrusive thoughts in Major Depressive Disorder (MDD) using wearable heart rate monitors. The study involved 18 participants meeting diagnostic criteria for a current major depressive episode and 18 healthy controls. Participants were assessed for baseline levels of various sociodemographic lifestyle questionnaires to measure levels of hostility, trait anxiety, depression, loneliness, and perseverative cognition. Three forms of perseverative cognition were measured: (1) depressive rumination using the RRS [38], which measures self-focused responses to depressed mood (2) worry using the Penn State Worry Questionnaire (PSWQ) [84], which captures anxiety about future outcomes and (3) reactive rumination using the Stress-Reactive Rumination Scale (SRRS) [85,86], which evaluates the tendency to ruminate following stressful events independent of depressive symptoms. Thereafter, participants wore chest-strapped HR monitors RS-800CX and Bodyguard 2 for 24 h while completing electronic diary entries every 30 min, reporting on their thoughts and emotions. Each diary entry assessed their thought content, duration, emotional valence, repetitiveness, and temporal focus (past/present/future), along with how much these thoughts interfered with activities and any attempts to suppress them. Participants also reported on factors that may affect heart rate, such as recent stressful events since the last entry, physical state (including posture, activity, and substance consumption), and current levels of mood across six dimensions: tiredness, anxiety, sadness, happiness, anger, and boredom. Thoughts were then categorized into three distinct classifications based on their content, valence, temporal dimension, and frequency of occurrence: being on task, mind wandering, and ruminating or worrying (PC). Additionally, in the morning, participants were instructed to complete the PROMIS sleep disturbance short form, and at night, before bed, they were instructed to complete the patient health questionnaire (PHQ-15) to assess somatic symptoms. Results revealed that PC produced distinct physiological changes that are detectable through wearable devices across both clinical and non-clinical populations. Specifically, participants with MDD and healthy controls showed significant reductions in HRV during periods of perseverative cognition [F(2,562) = 12.28; *p* < 0.0001], with MDD participants exhibiting higher heart rates and lower HRV overall [F(1,562) = 12.68; *p* < 0.0001]. This study was the first to combine Ecological Momentary Assessment (EMA) through electronic diaries with simultaneous ambulatory HR and HRV monitoring in individuals with MDD. This approach represents a significant advancement over traditional retrospective self-reporting methods by providing continuous, objective physiological measurements. Moreover, these findings demonstrate the potential for using wearable devices that could identify and alert users to ruminative episodes based on their HRV patterns.

The authors of [69] investigated the use of wearable physiological monitors to predict the presence and severity of suicidal thoughts, a specific form of rumination, in psychiatric inpatients. The study involved 25 suicidal psychiatric inpatients (14 female, 11 male) who were monitored during their inpatient stay (mean length 7 days) and for 28 days post-discharge. Upon enrollment, participants completed baseline assessments, including demographic questionnaires, history of suicidal thoughts and behaviors, and other trait-level measures. The study protocol combined continuous physiological monitoring with EMA. Participants were instructed to use an Empatica Embrace 2 wrist-worn wearable device 24 h per day (except during activities where the device could get wet) and completed six daily smartphone-based surveys, randomly distributed between 9 am and 11 pm. The surveys assessed suicidal thinking using a three-item measure adapted from the Beck Scale for Suicidal Ideation [87]. Items were rated on a 0–10 Likert scale evaluating (1) urge to die by suicide, (2) intention to kill oneself in the next day, and (3) ability to resist the urge to die by suicide. Researchers collected continuous EDA data as a measure of autonomic arousal through the wrist-worn device while simultaneously gathering EMA self-report data through the smartphone app. Results revealed that variability in EDA arousal was associated with periods of suicidal thinking, although models using physiological data alone had weaker predictive power, including those with self-report data. The combination of EDA data with self-report measures, particularly those capturing fewer overlapping constructs such as low-EDA-arousal and negative affect scores, significantly improved the prediction of suicidal thinking severity, as indicated by lower AIC (237.58) and BIC (259.43) and higher Bayes Factors (1023.02). Less overlapping constructs refers to the pairing of EDA data, which is more sensitive to high-arousal states like anger or agitation, with self-report measures that focus on low-arousal negative affect, such as feelings of hopelessness or burdensomeness. The improvement in prediction was evident in models incorporating low-arousal negative affect, likely because EDA, being more sensitive to high-arousal states, provided unique information about distress not captured by those self-report measures alone. Additionally, researchers found that approximately 75% of autonomic events occurred outside self-report times, highlighting the potential of wearables for identifying rumination episodes missed by traditional assessment methods. These findings demonstrate that physiological data from wearables can enhance the assessment of suicide related rumination severity when combined with carefully selected self-report measures that complement rather than duplicate physiological data.

The authors of [71] conducted a case study examining psychological, psychophysiological, and behavioral markers leading up to a suicide attempt, focusing on identifying in-the-moment indicators of acute suicidal thinking and risk. The study involved a single female participant in her 50s with borderline personality disorder who was monitored for seven days during their inpatient stay. Upon signing the informed consent, the participant completed baseline assessments, including demographic information, suicidal thought and behavior history, and trait-level measures. The study protocol combined continuous physiological monitoring with EMA. The participant wore an Empatica E4 wrist-worn wearable that captured EDA SCL, EDA SCR, and movement data via a three-axis accelerometer. Additionally, she completed six daily smartphone-based surveys delivered at random times within predefined windows. These surveys assessed current suicidal thoughts using a three-item measure adapted from the Beck Scale for Suicidal Ideation. The items were rated 0–9, evaluating (1) current desire to die by suicide, (2) current intention to die by suicide, and (3) current ability to resist the urge to die by suicide. The surveys also measured 15 momentary affective states based on PANAS, rated on a 1–10 scale. Researchers collected continuous SCL, SCR, and accelerometer data while simultaneously gathering self-report responses through smartphone surveys. Results revealed distinctive patterns in both physiological and behavioral data leading up to the participant’s suicide attempt on day 5. Specifically, during the four days preceding the attempt, researchers observed progressively increasing morning physical activity levels, SCL and SCR, with all physiological measures reaching their peak on day 4, the day before the attempt. Moreover, these physiological changes coincided with self-reported survey responses of suicidal thoughts and persistent negative affect. Although the graphs of average daily movement, SCL, and SCR visually demonstrated these physiological and behavioral patterns, no formal statistical analyses were conducted to examine the significance of these relationships. The temporal relationship between elevated EDA measurements and increased self-reported suicidal thinking demonstrates the potential value of continuous physiological monitoring for detecting acute suicide risk. These findings suggest that wearable devices capturing real-time physiological data, particularly EDA, may help identify periods of elevated suicidal thoughts, a form of rumination that might be missed by traditional assessment methods alone.

#### 3.4.3. Rumination in the Context of Ecological-Based Mindfulness

Ref. [70] investigated the cognitive and neurological effects of resting versus light physical activity in natural environments, examining how connectedness to nature and state-based mindfulness influences the outdoor experience, with particular attention to neural patterns associated with rumination. However, no formal rumination scales or measures were included in this study. The study involved 50 participants (15 female, 35 male) who were randomly assigned to either walking or sitting conditions in a campus green space. Prior to the intervention, participants completed baseline assessments, including surveys of daily routines, the 14-item Connectedness to Nature Scale, and five practice runs of the Stroop test to establish baseline cognitive performance. The intervention protocol involved 10 min sessions of either walking or sitting in a park-like quad containing grass, trees, and concrete paths. During the intervention, participants wore Emotiv Insight Portable EEG headsets measuring multiple brain wave patterns (frontal theta and beta, alpha, and gamma), along with fitness trackers monitoring HR. State mindfulness was assessed using the 21-item State Mindfulness Scale. Cognitive performance was measured using the EncephelApp^®^ Stroop (https://encephalapp.com/) task administered via smartphones at three time points: before, immediately after, and 10 min post-intervention. The study collected EEG measurements across different wave bands, including frontal beta activity, a potential indicator of rumination, HR data from fitness trackers, cognitive performance scores from the Stroop task, and self-report measures of nature connectedness and state mindfulness. Results found that participants with stronger nature connections and higher state-based mindfulness demonstrated lower levels of frontal beta activity during the outdoor intervention. Previous research has established that elevated frontal beta activity during rest can indicate rumination and heightened cognitive activity [5]. While this study did not explicitly examine the statistical relationship between EEG brain waves and rumination, the observed decrease in frontal beta activity during the mindfulness-based intervention suggests a potential reduction in ruminative thinking. This finding indicates that EEG monitoring, particularly of frontal beta wave activity, could serve as an objective biomarker for measuring rumination in natural settings. These findings highlight the potential of combining physiological measurements with environmental interventions to better understand and potentially reduce rumination patterns.

### 3.5. Measures of Rumination Across Studies

As shown in Table 4, the studies in our corpus used varied approaches to measure and induce rumination, specifically targeting negative ruminative thinking. In this section, we present the three primary measurement strategies that emerged: standardized scales, experimental induction protocols, and simplified self-report measures.

Standardized self-report scales were employed in several studies to assess rumination tendencies. Refs. [65,72] used the RRS [38], which measures depressive rumination through 22 items rated on a four-point Likert scale (1 = rarely to 4 = almost always). The RRS includes items such as “think about how alone you feel” and “think about all your shortcomings, failures, faults, mistakes”, explicitly targeting negative self-focused thoughts. Additionally, Ref. [65] incorporated the Stress-Reactive Rumination Scale (SRRS), which assesses trait rumination following stressful situations through 25 items rated on a four-point Likert scale [85,86]. The SRRS provides a trait rumination score between 0 and 250, with higher scores indicating higher traits [88]. The SRRS comprises three subscales: Negative Inferential Style, which assesses the tendency to ruminate about the potential impact of a stressor across various aspects of their life (e.g., “Ruminate about how the stressor will affect other areas of your life”), Hopelessness, which assesses the tendency to ruminate on the possibility of things never improving (e.g., “Think about the possibility that things will never get better”), and Active Problem-Solving, the tendency to identify solutions or opportunities for growth within challenging situations (“Try to find something positive in the situation or something you’ve learned”) [85,86]. Moreover, ref. [66] used the Mini-Cambridge-Exeter Repetitive Thought Scale (mini-CERTS) [77], a 16-item scale that assesses trait rumination on a 1–4 scale (1 = almost never, 4 = almost always). The mini-CERTS comprises two subscales: “concrete, experiential thinking”, a constructive form of rumination characterized by a mode of thinking centered on how one is presently feeling and experiencing the ongoing situation (e.g., I can grasp and respond to changes in the world around me without having to analyze the details) and “abstract, analytical thinking”, an unconstructive form of rumination consists in abstract thinking about the causes and consequence of one’s mood or condition (e.g., My thinking tends to spiral out from one specific event to broader, general aspects of my life). The authors of [66] also used four visual analog scales to measure momentary rumination rated on a scale of 0–100. These scales assessed different aspects of rumination: thoughts about feelings (VAS “Feelings”: “At this moment, I am thinking about my feelings”), focus on problems (VAS “Problems”: “At this moment, I am brooding about negative things”), brooding about negative things (VAS “Brooding”: referred to as VAS “Brooding”), and self-focus (VAS “Focused”: “At this moment, I am focused on myself”).

Experimental induction protocols were implemented in three studies, all adapting Nolen-Hoeksema and Morrow’s [78] rumination induction paradigm. The original paradigm was designed to influence thought patterns through 45 self-focused prompts administered over an 8 min period. Participants were instructed to “focus their attention on thoughts that were symptom-focused, emotion-focused, and self-focused”, with prompts such as “think about what your feelings might mean”, “the physical sensations you feel in your body”, and “how quick/slow your thinking is right now.” These prompts were intentionally designed to be emotionally neutral. Supported by many studies [78,89,90,91,92]. Nolen-Hoeksema theorized that because dysphoric individuals tend to have more negative feelings and thought patterns, their ruminative self-focus would naturally lead them to become significantly more dysphoric even in response to neutral self-focused prompts. This approach eliminates the need for explicitly negative content while maintaining ethical considerations for long-lasting effects of rumination induction [1]. Three studies in our review adapted this foundational paradigm with distinct modifications. Ref. [66] streamlined the protocol by first inducing temporary dysphoria through a deliberately impossible IQ task, then condensing the 45 prompts into one sentence, asking participants to “reflect upon the causes and consequences of their feelings” for five minutes. Ref. [73] combined the rumination induction paradigm with Cyberball, a task that induces temporary dysphoria through simulated social rejection. Since they were interested in comparing imagery-based rumination and verbal-based rumination, they incorporated specific language prompts for each condition. To distinguish between conditions, participants in the imagery-based group were instructed to “use your imagination” and “make mental images”, with prompts beginning with “Imagine”. Conversely, participants in the verbal-based group were guided to “use your concentration” and “make sentences”, with prompts starting with “Think about”. Throughout both conditions, the researchers maintained the self-focused nature of the original prompts, ensuring consistency with the rumination induction paradigm while clearly differentiating between the two cognitive processes under study. The authors of [67] combined the rumination induction with a TSST involving public speaking and mental math while receiving intentionally negative feedback from the experimenter to induce temporary dysphoria. Following this stressor, participants assigned to the rumination condition were asked to read and provide written responses to 10 “Why?” prompts (e.g., “Why am I feeling the level of motivation I feel right now?”). The researchers began each prompt with ‘Why?’ to encourage rumination, prompting participants to analyze and question the reasons behind their experiences. This approach, along with other adaptations, maintained the core theoretical foundation of Nolen-Hoeksema’s rumination induction paradigm, which proposes that inducing temporary dysphoria followed by self-focused attention would naturally elicit negative rumination patterns in participants.

Simplified self-report measures represented the third approach. The authors of [68] used a binary moment indicator specifically targeting racial injustice rumination, asking, “Over the course of the day, did you think about racial injustices and the mistreatment of people of color in the US?”. The authors of [69,71] used a three-item scale adapted from the Beck Scale for Suicidal Ideation to assess suicidal thinking in the present moment. On a scale of 0–10 scale (from “not at all” to “very strong”), the three items assessed the strength of (1) urge to die by suicide (i.e., “How intense is your desire to kill yourself right now?”), (2) the intention to kill oneself at some point during the next day (i.e., “How strong is your intention to kill yourself right now?”), and (3) the ability to resist the urge to die by suicide (i.e., “How strong is your ability to resist the urge to kill yourself right now?”).

Across all measurement approaches, the focus was predominantly on negative rumination patterns. Even when using neutral prompts in the rumination induction paradigm, the protocols typically included preliminary negative mood induction, leveraging the tendency of dysphoric individuals to ruminate negatively given neutral prompts [1,90]. This consistent focus on negative rumination patterns is evident throughout our corpus, whether through explicit scale items or experimental protocols. The field heavily draws from Nolen-Hoeksema’s pioneering work, with her RRS and rumination induction paradigm serving as gold standard assessment tools. This methodological consistency is particularly valuable in the emerging field of wearable sensor-based rumination detection, where researchers can anchor their physiological measurements to these validated psychological instruments. The widespread adoption of the RRS and its variants, along with experimental protocols based on Nolen-Hoeksema’s paradigm, provides a robust foundation for investigating rumination’s physiological correlates through wearable technology.

### 3.6. Comparative Validity of Biomarkers in Rumination Detection

Of the nine studies reviewed, seven directly assessed physiological biomarkers in relation to rumination. EDA emerged as a frequently studied (*n* = 5) and robust marker, with dynamic causal modeling revealing significantly elevated arousal during rumination in individuals with high anger and anxiety traits (*p* = 0.012, *p* = 0.006). EDA SCL was associated with rumination, showing delayed stress recovery (mean difference = 1.54 microsiemens, *p* = 0.01) and heightened levels during perseverative cognition. HRV showed significant reductions during rumination in individuals with MDD [F(1,562) = 12.68; *p* < 0.0001], though other studies provided qualitative rather than quantitative data on HRV changes. EEG frequency coherence showed reductions in overall brain coherence across multiple bands (theta, alpha, beta, gamma; [F(1,90) = 9.445, *p* = 0.003]) with an increase in coherence specifically in beta (t = 2.301, *p* = 0.017) and gamma (t = 2.411, *p* = 0.018) bands during rumination. These findings suggest that EDA, particularly SCL, may be a promising biomarker for identifying rumination, with HRV and EEG providing additional insights, though inconsistent statistical methods across studies make it difficult to determine which biomarker has the greatest true comparative validity. Additional research is needed to validate these patterns and explore contextual moderators such as individual traits and stress conditions.

## 4. Discussion

This scoping review has synthesized the existing research on wearable technology for detecting cognitive rumination and examined the sensors and wearable devices used, physiological biomarkers measured, standard measures of rumination used, and the comparative validity of specific biomarkers in identifying cognitive rumination. The review identified nine studies that used wearable devices to measure rumination-related physiological responses and biomarkers, revealing distinct physiological signatures associated with ruminative states across multiple measurement modalities.

Key findings revealed that EDA sensors measuring electrodermal activity emerged as both the most prevalent sensor and most comparatively valid biomarker for detecting cognitive rumination via wearable devices. EDA, derived from SCL and SCR, was used in our corpus. However, comparing these sub-measures proved challenging due to the use of varying statistical methods across studies. Consequently, it remains unclear which sub-measure of EDA is more reliable or has greater comparative validity for measuring cognitive rumination. Nonetheless, multiple studies in our corpus demonstrated significant associations between EDA and ruminative states. The authors of [67] found that individuals in rumination conditions maintained significantly elevated SCL during stress recovery, with differences persisting 30 min post-stressor compared to control groups. In a clinical application, ref. [71]’s case study documented a progressive increase in morning SCR and SCL measurements over four days preceding a participant’s suicide attempt, suggesting potential early warning capabilities. The authors of [69] further demonstrated that integrating EDA data with self-reported negative affect significantly enhanced the accuracy of suicidal ideation prediction models. Adding contextual insight, ref. [68] using dynamic causal modeling revealed that individuals with elevated anger and anxiety traits showed significantly increased EDA arousal during periods of race-related rumination, as documented through daily diary entries. EEG sensors measuring brain activity were the second most prevalent sensor and comparatively valid biomarker. The authors of [72]’s between-group EEG analysis revealed increased coherence in beta and gamma frequency bands, which previous research has shown may indicate reduced control over intrusive thoughts and potentially stem from excessive self-focused depressive states [81] induced by the NLP-based rumination induction questionnaire [82,83]. Complementing these findings, ref. [70] identified that participants reporting stronger nature connection and higher state-based mindfulness showed reduced frontal beta activity. In contrast, they note that previous research has found that elevated frontal beta activity during rest can indicate rumination and heightened cognitive activity [5]. HRV emerged as the third most comparatively valid biomarker, measured using both commercial heart rate monitors and wearable ECG devices. The authors of [65] documented significant HRV reductions during perseverative cognition periods in both MDD patients and healthy controls. Similarly, ref. [73] found reduced HF-HRV levels in participants who were assigned to the rumination induction condition compared to those assigned to the distraction condition. Moreover, researchers used EMG sensors to measure muscle activity and accelerometers to track movement, though these were less prevalent. The authors of [66]’s study revealed that even during silent rumination tasks, participants showed increased activity in speech-related muscles (including the lips, forehead, and jaw) when engaging in internal dialogue. This physiological evidence supports the connection between inner speech and rumination. The Empatica E4 and Embrace 2 wrist-worn devices emerged as the most used wearable devices, with wireless EEG headsets being the second most prevalent devices used. The RRS, developed by Nolen-Hoeksema and colleagues [38], emerged as the predominant standardized measure for assessing rumination. Researchers frequently employed experimental induction protocols, primarily adaptations of the rumination induction paradigm established by Nolen-Hoeksema and Morrow [78], to elicit ruminative states in participants.

Studies in our corpus explored rumination in diverse contexts, including its role in depression and suicidal ideation in clinical populations [65,69,71], the impact of race-related stress on minority students [68], and the interplay of rumination, stress recovery, and mindfulness in everyday life [66,67,70,72,73]. This review demonstrated that wearable devices can effectively capture real-time physiological responses associated with rumination, including elevated EDA, reduced HRV, EMG changes, and alterations in brainwave coherence patterns. The combination of different physiological markers, especially when integrated with traditional self-report measures and EMA via smartphone diaries, offers promising opportunities for more comprehensive rumination detection and monitoring.

### 4.1. Opportunities and Challenges

While wearable devices demonstrate significant potential in capturing real-time physiological responses associated with rumination, their implementation presents both promising opportunities and notable challenges that warrant careful consideration. The opportunities stem primarily from these devices’ ability to provide continuous, objective measurement in real-world settings, offering unique advantages over traditional assessment methods. This continuous monitoring capability has proven particularly valuable, as demonstrated by [69]’s discovery that approximately 75% of autonomic events occurred between scheduled self-report windows. This finding highlights how wearables can capture ruminative states that traditional, time-bound assessment methods might miss. Building on this advantage, ref. [67]’s research revealed that wearables can detect physiological stress responses even when individuals are not consciously aware of their ruminative state, offering valuable insights into unconscious rumination patterns. However, our analysis reveals that the most effective approach to rumination detection currently combines physiological data with digital self-report measures, such as adapted versions of the RRS or electronic diaries. These self-report assessments provide the necessary context for researchers and clinicians to distinguish ruminative states from other overlapping forms of anxiety and stress [93,94]. That said, their implementation must be judicious to avoid questionnaire fatigue and ensure sustained user engagement [45,95]. The integration of physiological data with traditional self-report assessment methods has further enhanced the utility of wearable devices. The authors of [69]’s research demonstrated this synergy, finding that while models relying solely on physiological data had limited predictive capability, those combining physiological data with self-report measures significantly improved the accuracy of rumination prediction in the context of suicidal thinking severity. Similarly, ref. [68]’s research showed how wearable sensors, in combination with self-report measures, can capture complex contextual influences on autonomic arousal, such as the interaction between predisposed anger and anxiety traits during racism-related rumination. Despite these promising applications, several significant challenges emerge in the practical implementation of these technologies. Data quality and user compliance represent primary concerns, as illustrated by [68]’s finding that participants found wrist-worn devices stiff and uncomfortable, leading to intermittent usage breaks that compromised data continuity and quality. Technical limitations present additional barriers, particularly regarding proprietary algorithms in research-grade devices like the Empatica wrist-worn devices. These algorithms, owned and protected by specific entities, require licensing for use and operate with undisclosed methodologies, potentially hindering research replication and validation efforts. Given these considerations, the implementation of wearable technology in rumination research and clinical applications requires carefully balancing their substantial benefits against their technical and practical challenges.

### 4.2. Wearables in the Broader Context of Rumination Research

Recent advances in wearable technology for measuring physiological biomarkers have enabled new approaches to detecting and monitoring rumination in naturalistic settings, though important gaps remain between laboratory-established physiological markers and current wearable device measurement capabilities. To note, this review focused specifically on negative rumination characterized by perseverative negative thinking rather than broader categories like intrusive thoughts or reflective rumination, which may require different detection approaches. Moreover, research indicates that individuals commonly ruminate about distinct types of negative content, including interpersonal relationships, past mistakes, and social interactions [37]. These variations in rumination content may correlate with or be better detected by particular biomarkers, a critical consideration for designing rumination detection algorithms and sensor systems. Our findings highlight three primary sensor-based measurement modalities, EDA, EEG, and HRV, complemented by EMG and Accelerometer sensors. These measurement techniques are grounded in well-established laboratory research indicating the presence of relationships between rumination and autonomic nervous system activity. For instance, ref. [96] found increased galvanic skin response following rumination induction in highly anxious women, while [3] observed decreased HF-HRV during induced rumination in individuals with low trait rumination. A meta-analysis by [17] established that perseverative cognition, which includes rumination, is consistently associated with autonomic dysregulation, particularly decreased HRV and increased cardiovascular activation. This growing understanding of autonomic nervous system involvement in rumination [15] helps explain the prominence of EDA and HRV measurements in the wearable studies presented in our corpus. The successful implementation of these measurements in wearable form suggests promising potential for continuous monitoring of rumination in naturalistic settings. Several studies in our review have begun to realize this potential for ecological monitoring. For instance, ref. [65] demonstrated the feasibility of 24 h heart rate monitoring combined with electronic diary entries, while [69] achieved continuous physiological monitoring of psychiatric inpatients for extended periods, including post-discharge follow-up. These studies represent significant advances toward ecological validity in rumination detection, moving beyond traditional laboratory-based assessments. However, our review reveals important gaps between laboratory-established physiological correlates and current wearable measurement capabilities. The broader literature has identified several additional physiological concomitants of rumination that are not yet widely implemented in wearable technology [16]. For example, ref. [97] found that inducing rumination led to increased systolic blood pressure, while [98] demonstrated associations between rumination and elevated cortisol levels in response to laboratory stressors. Recent technological advances may help bridge these gaps. New developments in wearable cortisol patches [99,100] and improved continuous blood pressure monitoring devices [101,102] present opportunities for more comprehensive physiological monitoring of rumination. For example, emerging sensors might eventually enable precise continuous blood pressure monitoring through wearable devices, while advances in sweat sensing technology could potentially enable non-invasive cortisol monitoring.

Several promising directions for future research emerge from our findings. First, the development of multimodal sensing approaches could improve the specificity of rumination detection. While single biomarkers like EDA or HRV show promise, combining multiple physiological signals might better differentiate rumination from other cognitive–emotional states. Second, the prominence of EDA as a key biomarker for measuring rumination raises the question of whether EDA, SCL, SCR, or a combination of these measures is more reliable and valid for assessing real-time cognitive rumination. Third, advances in machine learning techniques could help identify complex patterns across multiple physiological parameters that might be uniquely indicative of ruminative states. Finally, future studies should build upon initial successes in longer-term monitoring protocols found in our corpus to examine temporal patterns in rumination, including its relationship with sleep, daily activities, and environmental contexts.

## Figures and Tables

**Table 1:** This table presents the databases and keywords used in the literature search.

Database	Search Strategies
IEEE	((“All Metadata”:wearable OR wearables OR “wearable device” OR “wearable devices” OR “wearable sensor*” OR “wearable electronic*” OR “wearable technolog*” OR “wearable computer*” OR “mobile sensor” OR “mobile sensors” OR “physiological monitoring” OR biometric* OR “heart rate monitor*” OR EEG OR “skin conductance” OR “skin conduction” OR “respiratory rate monitor*” OR “cortisol measur*”) AND (“All Metadata”:rumination OR ruminating OR ((perseverative OR perseveration OR intrusive OR negative) AND (thinking OR thoughts))) NOT (livestock OR cattle OR sheep OR cow OR dairy))
Scopus	((TITLE-ABS-KEY ((wearable OR wearables OR “wearable device” OR “wearable devices” OR “wearable sensor*” OR “wearable electronic*” OR “wearable technolog*” OR “wearable computer*” OR “mobile sensor” OR “mobile sensors” OR “physiological monitoring” OR biometric* OR “heart rate monitor*” OR eeg OR “skin conductance” OR “skin conduction” OR “respiratory rate monitor*” OR “cortisol measur*”))) AND (TITLE-ABS-KEY (rumination OR ruminating OR ((perseverative OR perseveration OR intrusive OR negative) AND (thinking OR thoughts))))) AND NOT (TITLE-ABS-KEY (livestock OR cattle OR sheep OR cow OR dairy))
PubMed	((“Wearable Electronic Devices”[Mesh] OR wearable[tw] OR wearables[tw] OR “wearable device”[tw] OR “wearable devices”[tw] OR “wearable sensor*”[tw] OR “wearable electronic*”[tw] OR “wearable technolog*”[tw] OR “wearable computer*”[tw] OR “mobile sensor”[tw] OR “mobile sensors”[tw] OR “physiological monitoring”[tw] OR biometric*[tw] OR “heart rate monitor*”[tw] OR EEG[tw] OR “skin conductance”[tw] OR “skin conduction”[tw] OR “respiratory rate monitor*”[tw] OR “cortisol measur*”[tw])) AND (“Rumination, Cognitive”[Mesh] OR rumination[tw] OR ruminating[tw] OR ((perseverative[tw] OR perseveration[tw] OR intrusive[tw] OR negative[tw]) AND (thinking[tw] OR thoughts[tw]))) NOT (livestock[tw] OR cattle[tw] OR sheep[tw] OR cow[tw] OR dairy[tw])
PsycInfo Database	(MAINSUBJECT.EXACT.EXPLODE(“Wearable Devices”) OR wearable OR wearables OR “wearable device” OR “wearable devices” OR “wearable sensor*” OR “wearable electronic*” OR “wearable technolog*” OR “wearable computer*” OR “mobile sensor” OR “mobile sensors” OR “physiological monitoring” OR biometric* OR “heart rate monitor*” OR EEG OR “skin conductance” OR “skin conduction” OR “respiratory rate monitor*” OR “cortisol measur*”) AND (MAINSUBJECT.EXACT.EXPLODE(“Rumination (Cognitive Process)”) OR MAINSUBJECT.EXACT.EXPLODE(“Perseveration”) OR rumination OR ruminating OR ((perseverative OR perseveration OR intrusive OR negative) AND (thinking OR thoughts))) NOT noft(livestock OR cattle OR sheep OR cow OR dairy) Search Strategies
PubMed	((“Wearable Electronic Devices”[Mesh] OR wearable[tw] OR wearables[tw] OR “wearable device”[tw] OR “wearable devices”[tw] OR “wearable sensor*”[tw] OR “wearable electronic*”[tw] OR “wearable technolog*”[tw] OR “wearable computer*”[tw] OR “mobile sensor”[tw] OR “mobile sensors”[tw] OR “physiological monitoring”[tw] OR biometric*[tw] OR “heart rate monitor*”[tw] OR EEG[tw] OR “skin conductance”[tw] OR “skin conduction”[tw] OR “respiratory rate monitor*”[tw] OR “cortisol measur*”[tw])) AND (“Rumination, Cognitive”[Mesh] OR rumination[tw] OR ruminating[tw] OR ((perseverative[tw] OR perseveration[tw] OR intrusive[tw] OR negative[tw]) AND (thinking[tw] OR thoughts[tw]))) NOT (livestock[tw] OR cattle[tw] OR sheep[tw] OR cow[tw] OR dairy[tw])
IEEE Scopus	((“All Metadata”:wearable OR wearables OR “wearable device” OR “wearable devices” OR “wearable sensor*” OR “wearable electronic*” OR “wearable technolog*” OR “wearable computer*” OR “mobile sensor” OR “mobile sensors” OR “physiological monitoring” OR biometric* OR “heart rate monitor*” OR EEG OR “skin conductance” OR “skin conduction” OR “respiratory rate monitor*” OR “cortisol measur*”) AND (“All Metadata”:rumination OR ruminating OR ((perseverative OR perseveration OR intrusive OR negative) AND (thinking OR thoughts))) NOT (livestock OR cattle OR sheep OR cow OR dairy)) ((TITLE-ABS-KEY ((wearable OR wearables OR “wearable device” OR “wearable devices” OR “wearable sensor*” OR “wearable electronic*” OR “wearable technolog*” OR “wearable computer*” OR “mobile sensor” OR “mobile sensors” OR “physiological monitoring” OR biometric* OR “heart rate monitor*” OR eeg OR “skin conductance” OR “skin conduction” OR “respiratory rate monitor*” OR “cortisol measur*”))) AND (TITLE-ABS-KEY (rumination OR ruminating OR ((perseverative OR perseveration OR intrusive OR negative) AND (thinking OR thoughts))))) AND NOT (TITLE-ABS-KEY (livestock OR cattle OR sheep OR cow OR dairy))

**Table 2:** Presents an at-a-glance summary of the selected studies. This summary includes details such as the article title, the brand and name of the wearable device used, the type of sensor employed, the biomarker data collected, any additional assessments used to measure rumination, the aim of the study, the sample size, and the corresponding references.

Article Title	Devices	Sensor Type	Biomarkers	Additional Assessments	Aim of Study	Sample Size	Reference
Cognitive, behavioral, and autonomic correlates of mind wandering and perseverative cognition in major depression	RS-800CX (Polar Electro, Guangzhou, China) and Bodygaurd 2 (Firstbeat, Jyväskylä, Finland)	HR	HRV	Self-Report Scale and Electronic Diary	Examines the effects of different types of mind wandering, including rumination, on mood and autonomic function in Major Depressive Disorder	36	Ottaviani et al. [65]
Orofacial electromyographic correlates of induced verbal rumination	Trigno Mini sensors and Trigino wireless EMG system (Delsys Inc., Natick, MA, USA)	EMG	Muscle Response	Self-Report Scale	Examines physiological correlates of verbal rumination, specifically facial and non-facial muscle activity during rumination	72	Nalborczyk et al. [66]
Worry and rumination: do they prolong physiological and affective recovery from stress?	Neulog GSR logger sensor NUL-217 (NeuLog)	GSR	Skin Conductance	Self-Report Scale	Examines the effect on physiological recovery from stress of worry and rumination	54	Capobianco et al. [67]
Do Trait Psychological Characteristics Moderate Sympathetic Arousal to Racial Discrimination Exposure in a Natural Setting?	Empatica E4 (Empatica, Boston, MA, USA)	EDA	Skin Conductance	Electronic Diary	Examines how psychological health traits moderate the relationship between various forms of race-related stress (including discrimination and rumination) and sympathetic nervous system arousal, using wearable sensors to measure participants’ responses in daily life	100	Jelsma et al. [68]
Can passive measurement of physiological distress help better predict suicidal thinking?	Empatica Embrace 2 (Empatica, Boston, MA, USA)	EDA	Skin Conductance	Self-Report Scale and EMA	Examines whether EDA can help predict the presence of suicidal thinking and/or severity of suicidal thinking beyond self-report	25	Kleiman et al. [69]
Walking and Sitting Outdoors: Which Is Better for Cognitive Performance and Mental States?	Emotiv Insight portable headset (Emotiv Inc.)	EEG	Brain Activity		Compares the cognitive and neurological effects of resting versus light physical activity in the same natural environment while also examining how connectedness to nature and state-based mindfulness influence the outdoor experience	50	Bailey and Kang [70]
Real-Time Digital Monitoring of a Suicide Attempt by a Hospital Patient	Empatica E4	EDA and Accelerator	Skin Conductance and Movement	Self-Report Scale and EMA	Examines real-time sensor data collected before, during, and after a patient’s suicide attempt during psychiatric hospitalization to identify psychological, psychophysiological, and behavioral markers of imminent suicidal behavior and thinking	1	Coppersmith et al. [71]
Questionnaires based on natural language processing elicit immersive ruminative thinking in ruminators: Evidence from behavioral responses and EEG data	Wireless EEG ZhenTec NT1 (Xi’an ZhenTec Intelligence Technology Co., Ltd., Xi’an, China)	EEG	Brain Activity	Self-Report Scale	Develops a rumination-inducing questionnaire using Natural Language Processing (NLP) and records EEG responses to this questionnaire, aiming to create a method for early detection of mental disorders in psychological screening processes	4591	Li et al. [72]
Reimagining rumination? The unique role of mental imagery in adolescent’s affective and physiological response to rumination and distraction	MindWare Mobile Acquisition Unit (MindWare Technologies, Ltd., Westerville, OH, USA)	ECG and EDA	HRV and Skin Conductance		Compares physiological responses (SCR and HF-HRV) to imagery-based and verbal rumination and distraction in adolescents, aiming to understand why imagery-based rumination may be more problematic and to inform assessment and intervention strategies for rumination	145	Lawrence et al. [73]

**Table 3:** This table references the device type, device brand and model name, and the number of studies that used the device.

Device Type	Device Model	Number of Studies
Heart Rate Monitor	RS-800CX (Polar Electro) and Bodyguard 2 (Firstbeat)	1
Wireless EMG Biofeedback System	Trigno Mini sensors and Trigno wireless EMG system	1
GSR Sensor	Neulog GSR logger sensor NUL-217	1
Wrist-worn	Empatica E4	3
	Empatica Embrace 2	
Wireless EEG Headset	Emotiv Insight portable headset	2
	Wireless EEG ZhenTec NT1	
Portable Mobile Health Monitor	MindWare Mobile Acquisition Unit	1

**Table 4:** This table references the types of rumination-related assessment or induction used for each study and their corresponding citation.

Rumination Assessment	Reference
Rumination Response Scale and Stress-Reactive Rumination Scale	[65]
Rumination Induction Prompt Mini-Certs and VAS scale	[66]
Rumination Induction Prompt	[67]
Self-Report Binary Moment Indicator	[68]
3-Item Suicidal Thinking Scale	[69]
None	[70]
3-Item Suicidal Thinking Scale	[71]
Rumination Response Scale	[72]
Rumination Induction Prompt	[73]

## Data Availability

No new data were created or analyzed in this study. Data sharing is not applicable to this article.
